# Overlap Between Gastroesophageal Reflux Disease and Irritable Bowel Syndrome and Its Impact on Quality of Life

**DOI:** 10.7759/cureus.50840

**Published:** 2023-12-20

**Authors:** Sulaiman A Alshammari, Mohsen N Almutairi, Mohammad O Alomar, Ziyad M Alsherif, Faisal H Alsubaie, Abdulrahman I Almezaini

**Affiliations:** 1 Family and Community Medicine, King Saud University College of Medicine, Riyadh, SAU

**Keywords:** saudi arabia, rome iv criteria, irritable bowel syndrome, gerd-hrql, gastroesophageal reflux disease

## Abstract

Background

Gastroesophageal reflux disease (GERD) and irritable bowel syndrome (IBS) are common gastrointestinal disorders that can negatively affect quality of life and healthcare costs. The co-occurrence of these conditions can lead to more complex symptomatology and therapeutic challenges. Therefore, understanding the extent of overlap between GERD and IBS is paramount. This study aims to estimate the overlap between GERD and IBS in Saudi Arabia and its impact on quality of life.

Methods

Patients with GERD at primary care clinics at King Khalid University Hospital in Riyadh, Saudi Arabia, were the subjects of cross-sectional research. The patients were selected using a simple random sampling technique, and an electronic questionnaire was utilized to collect data. Symptoms of IBS were assessed based on Rome IV criteria, and quality of life was evaluated using the GERD quality of life tool.

Results

Of the 293 GERD patients, the prevalence of co-occurrence of IBS among GERD patients was 35.8%. The GERD health-related quality of life (GERD- HRQL) scores ranged from 0 to 50, with a median and interquartile range (IQR) of 14 (8.5-20). Those with GERD and IBS had a significantly higher GERD-HRQL score than the patients with GERD alone (11 vs. 9, p-value: 0.049).

Furthermore, patients with GERD and IBS had a significantly higher GERD-HRQL score than the patients with IBS alone (15 vs. 11, p-value: 0.001). Of the total participants, 29.4% reported having abdominal pain in the last three months. The majority of the participants (55.6%) reported experiencing abdominal pain one to two times per week, while 22.2% reported experiencing it two to three days per month.

Conclusion

The high prevalence of IBS co-existence among GERD patients highlights the importance of considering both diseases in clinical practice to improve patient outcomes. The study also found that patients with both GERD and IBS had a significantly lower quality of life than those with GERD or IBS alone.

## Introduction

Gastroesophageal reflux disease (GERD) and irritable bowel syndrome (IBS) are prevalent gastrointestinal disorders that exert a significant influence on the well-being of individuals and impose substantial healthcare costs [1]. Current literature suggests that approximately 3.8% of the global population grapples with GERD [2], while IBS's worldwide incidence hovers between 5% and 10% among healthy individuals [3,4]. Common GERD symptoms encompass heartburn, although patients often present with additional complaints such as dysphagia, nausea, epigastric discomfort, belching, and odynophagia [5]. Conversely, typical IBS manifestations include abdominal pain, discomfort, alterations in bowel habits, and stool frequency [4]. The diagnostic journey for GERD and IBS relies primarily on clinical evaluation due to the absence of specific diagnostic tests [4,6]. Despite their distinct clinical manifestations, several studies have alluded to the potential co-occurrence of these conditions within the same patient [1,7]. If left untreated, GERD can give rise to complications such as Barrett's esophagus, esophageal erosion, ulceration, and stricture formation [8].

In contrast, complications associated with IBS span from constipation or diarrhea to conditions like hemorrhoids [4]. Recent evidence suggests a possible overlap between these conditions in certain patient populations. A recent study employing survey methods reported a GERD-IBS overlap ranging from 3% to 79%, while endoscopy-based investigations revealed a range of 10% to 74% [9]. Similarly, another study found that 34.4% of GERD-diagnosed patients exhibited concurrent IBS, and conversely, 62.4% of individuals with IBS were diagnosed with GERD [10]. Studies conducted in Saudi Arabia further underscore the prevalence of GERD and IBS, with reported rates of 45.4% and 31.8%, respectively [11,12].

Treatment options for patients experiencing this co-occurrence are primarily centered around lifestyle modifications, which include the consumption of soluble fiber, adopting a low FODMAP diet, avoiding trigger foods, weight loss in overweight and obese patients, and maintenance of gut microbiota [9,13,14]. Cognitive behavioral therapy also reduces the symptoms of both diseases through changes in cognitions (e.g., illness perception, catastrophizing, etc.), emotions, and behaviors [15,16]. Medical interventions predominantly involve medications that aim at alleviating abdominal pain, managing bowel movements, and neutralizing stomach acids, such as antispasmodics, proton pump inhibitors, and tricyclic antidepressants [13,14,17].

Consequently, identifying the coexistence of GERD and IBS might be critical, as it can guide clinicians in tailoring effective treatment strategies that could benefit affected individuals. In this context, our study aims to investigate the extent of co-occurrence between GERD and IBS, underscoring the clinical significance of recognizing their potential coexistence, which may sometimes be overlooked in routine clinical practice. Furthermore, our research aims to gauge the impact of these conditions on patients' health-related quality of life and how it evolves with a diagnosis of GERD, IBS, or both. This holistic understanding will heighten physicians' awareness of the potential interplay between these diseases, paving the way for more precise diagnoses, personalized treatment, and improved patient outcomes.

## Materials and methods

Study design

A cross-sectional study design was utilized with a sample of patients diagnosed with GERD from primary care clinics at King Khalid University Hospital (KKUH) in Riyadh, Saudi Arabia. The data collection was conducted from October 2022 to March 2023.

Study population

In this study, the participants were chosen using a simple random selection method from the population of patients who had been diagnosed with GERD and were receiving treatment at primary care clinics located at KKUH. The sample size calculation was performed utilizing the single proportion formula (N = (Za/2) 2 -1*P(1-P) / D^2) with a 95% confidence level and a 5% degree of precision. For Z=1.96, the minimum sample size was 311. The proportion used in this formula was taken from a systematic review of the same issue [18]. The phone numbers of all GERD patients at KKUH were collected as a sampling frame. A computer-generated random list of patient phone numbers was used as the sampling element.

Data collection

The data was acquired via an electronic survey instrument distributed through mobile phones. The phone numbers of the patients were obtained from the hospital's medical records. Symptoms of IBS were assessed based on Rome IV criteria, and their impact on quality of life was evaluated. The study was conducted over six months. The study utilized a structured questionnaire, which comprised four sections, including personal and demographic information, the Rome IV criteria for the IBS diagnosis tool [19], and the GERD quality of life tool [20]. These tools were validated and had been used in previous research. The GERD questionnaire, comprising six items, investigated the co-occurrence according to patients' reported symptoms. The diagnosis was considered for those with scores >8. The assessment of health-related quality of life was conducted utilizing a validated instrument consisting of 10 items. The responses to the GERD-HRQL were categorized into six grades: no symptoms = 0; symptoms noticeable, but not bothersome = 1; symptoms noticeable and bothersome, but not every day = 2; symptoms bothersome every day = 3; symptoms affect daily activities = 4; and symptoms are incapacitating, unable to do everyday activities = 5.

Statistical analysis

Statistical analysis was conducted using SPSS Statistics version 29.0 (IBM Corp. Released 2022. IBM SPSS Statistics for Windows, Version 29.0. Armonk, NY: IBM Corp). The variable of interest was the co-occurrence of IBS and GERD. In contrast, the secondary outcome variable GERD-HRQL was summed and tested for normal distribution via the Shapiro-Wilk test. The summary statistics for the total scores were computed using the median and interquartile range (IQR) due to skewed data and outliers. Frequency counts and percentages have described the categorical variables (sex, marital status, and BMI groups).

In comparing patients with GERD alone and those with IBS co-occurring with GERD, healthy cases were excluded from the analysis. The Mann-Whitney test (which is a nonparametric test used to compare differences between two independent groups when the dependent variable is not normally distributed) was employed to investigate a statistically significant difference in GERD-HRQL scores between the two groups, with the significance level set at 0.05.

Ethical considerations

All study participants voluntarily participated in the research without any incentives or rewards and were informed about the purpose of the study and their entitlement to decline participation or withdraw at any time. Furthermore, their privacy and confidentiality were maintained throughout the study. The research was approved by the Institutional Review Board (IRB) of King Saud University College of Medicine in Riyadh, Saudi Arabia, on October 25, 2022 (IRB approval of research project no. E-22-7339).

## Results

The study included 293 participants, and the response rate was 94.2%, with 53.6% being female and 46.4% being male. The age range of the participants was 18 to over 65, with the majority being between 30 and 44 years old. As for the participants' BMI, 38.6% were obese, while 25.6% had a normal BMI. Over half of the participants had a government job, while 33.1% were not working. The patients' characteristics are shown in Table 1.

**Table 1:** Sociodemographic characteristics of the study participants

n=293		N	%
Age	18-29	81	27.60%
	30-44	106	36.20%
	45-54	53	18.10%
	55-65	37	12.60%
	>65	16	5.50%
Sex	Female	157	53.60%
	Male	136	46.40%
Marital status	Single	92	31.40%
	Married	181	61.80%
	Divorced	14	4.80%
	Widow	6	2.00%
BMI groups	Underweight	11	3.80%
	Normal	75	25.60%
	Overweight	94	32.10%
	Obese	113	38.60%
Occupation	Not working	97	33.10%
	Governmental employee	111	37.90%
	Private sector employee	46	15.70%
	Student	39	13.30%
Income	<5,000	105	35.80%
	5,000-1,000	70	23.90%
	1,000-15,000	49	16.70%
	>1,5000	69	23.50%

Smokers represented 16.7%, while 15.4% reported exercising regularly. Most participants (59.4%) rated their sleep quality as medium. In terms of psychological stressors, 68.3% reported experiencing them sometimes, while 18.4% reported always experiencing them. The most common type of psychological stressor reported was personal and family issues (48.6%). Lifestyle and psychological characteristics are shown in Table 2.

**Table 2:** Lifestyle and psychological characteristics of the study participants

n=293		N	%
Smoking status	Yes	49	16.70%
	No	244	83.30%
Do you exercise?	Yes	45	15.40%
	No	248	84.60%
Quality of sleep	Bad	31	10.60%
	Medium	174	59.40%
	Good	88	30.00%
Do you face any emotional stressors?	Never	39	13.30%
	Sometimes	200	68.30%
	Always	54	18.40%
What is the most probable cause of that stress?	Personal and family issues	124	48.60%
	Health issues	37	14.50%
	Financial issues	32	12.50%
	Study issues	22	8.60%
	Work issues	40	15.70%

**Figure 1:**
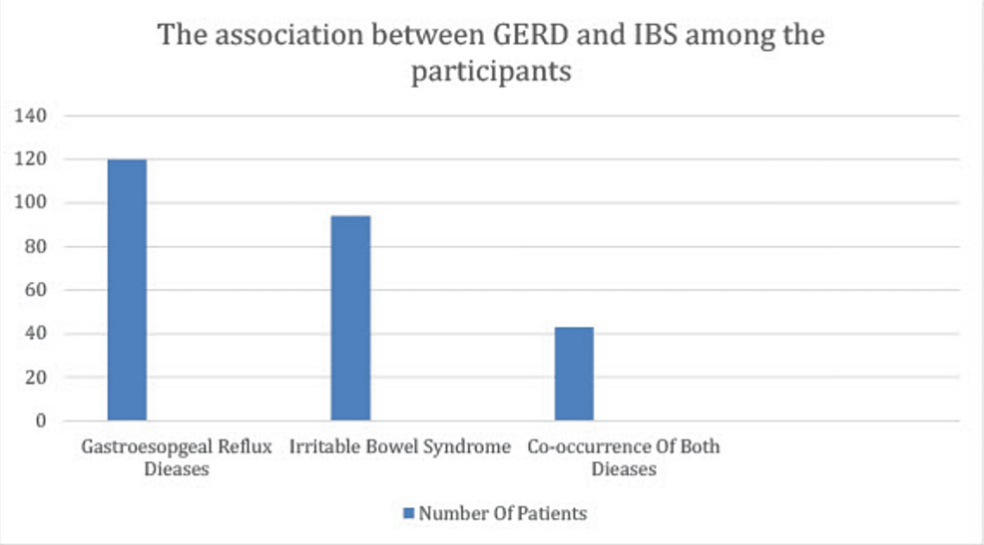
The co-occurrence of GERD and IBS GERD: gastroesophageal reflux disease, IBS: irritable bowel syndrome

Of all participants, 120 (40.9%) fulfilled the GERD questionnaire criteria. Among those who were categorized as diseased using the GERD questionnaire, 35.8% had overlapping IBS. However, this association between GERD and IBS was not statistically significant. Those with GERD and IBS had a significantly higher GERD-HRQL score than those with GERD alone (11 vs. 9, p-value: 0.049). Furthermore, patients with GERD and IBS had a significantly higher GERD-HRQL score than the patients with IBS alone (15 vs. 11, p-value: 0.001). Table 3 illustrates the overlap of GERD and IBS among the participants and GERD-HRQL scores for participants with GERD or IBS only and those with both GERD and IBS. The cooccurrence of GERD and IBS is presented in a bar diagram in Figure 1.

**Table 3:** The overlap between GERD and IBS among the participants and GERD-HRQL scores for participants with GERD or IBS only and those with both GERD and IBS IBS: irritable bowel syndrome, GERD: gastroesophageal reflux disease, GERD-HRQL: gastroesophageal reflux disease health-related quality of life questionnaire

A. The association between GERD and IBS						
			IBS		p-value	Odds ratio
			Yes	No		
GERD	Yes	120 (40.9%)	43 (35.8%)	77 (64.2%)	0.256	1.3-0.256
	No	173 (59.0%)	51 (29.5%)	122 (70.5%)		
B. GERD-HRQL among GERD and IBS versus GERD patients						
GERD-HRQL	GERD and IBS		Only GERD		p-value	
Median	11		9		0.049	
IQR	5-18		3-14			
Mean rank (Mann-Whitney)	161.2		140.3			
C. GERD-HRQL among GERD and IBS versus IBS patients						
GERD-HRQL	GERD and IBS		IBS		p-value	
Median	15		11		<0.001	
IQR	11-23		4-18			
Mean rank (Mann-Whitney)	103.9		76.3			

**Figure 2:**
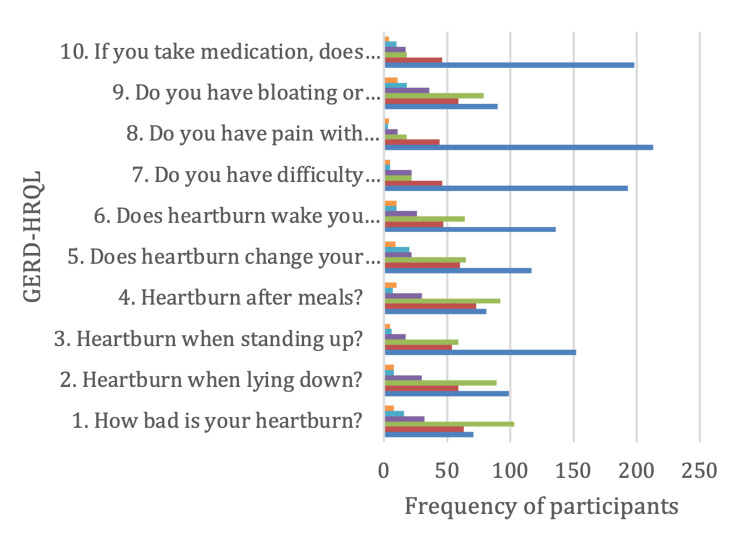
Items of GERD-HRQL as reported by the study participants GERD-HRQL: gastroesophageal reflux disease health-related quality of life, blue: no symptoms, red: symptoms noticeable, but not bothersome, gray: symptoms noticeable and bothersome, but not every day, orange: symptoms bothersome every day, sky blue: symptoms affect daily activities, green: symptoms are incapacitating, unable to do daily activities

The responses to the GERD-HRQL items were categorized into six grades, as shown in Figure 2: no symptoms = 0; symptoms noticeable, but not bothersome = 1; symptoms noticeable and bothersome, but not every day = 2; symptoms bothersome every day = 3; symptoms affect daily activities = 4; and symptoms are incapacitating, unable to do everyday activities = 5. The GERD-HRQL scores ranged from 0 to 50 with a median and IQR of 14 (8.5-20).

Employing Rome IV criteria for IBS, 29.4% of the participants reported having abdominal pain in the last three months. The majority of the participants (55.6%) reported experiencing abdominal pain shortly before, during, or shortly after having a bowel movement one to two times per week, while 24.2% reported experiencing it two to three days per month. Additionally, 50.7% reported experiencing stool that was softer or harder with the pain, and 53.6% stated that bowel movements became either more frequent than usual or less frequent than usual when they experienced this pain. Finally, it was found that 80.7% of the study participants had experienced abdominal pain for less than six months. The details of the Rome IV criteria for IBS are shown in Table 4.

**Table 4:** Rome IV criteria describing the occurrence and intensity of IBS symptoms

N=293		N	%
Have you suffered from abdominal pain in the last 3 months?	Yes	86	29.40%
	No	207	70.60%
How many times have you suffered from abdominal pain in the last three months?	2 or less days/3 months	38	18.40%
	1 day/month	28	13.50%
	2-3 days/month	46	22.20%
	1 day/week	15	7.20%
	2-3 days/week	44	21.30%
	4-6 days/week	20	9.70%
	Everyday	16	7.70%
How many times did you experience this pain in your abdomen shortly before, during, or shortly after having a bowel movement?	None	42	20.30%
	1-2 times/month	115	55.60%
	>2 times/month	50	24.20%
How often did your stools become either softer than usual or harder than usual when you experienced this pain?	None	27	13.00%
	1-2 times	105	50.70%
	>2 times	75	36.20%
How many times did your bowel movements become either more frequent than usual or less frequent than usual when you experienced this pain?	None	43	20.80%
	1-2 times	111	53.60%
	>2 times	53	25.60%
Has it been 6 months or longer since you started having this pain?	Yes	40	19.30%
	No	167	80.70%

Regarding GERD, 15.4% and 14.7% of the participants reported a burning feeling behind their breastbone and food or water going up to their throat four to seven days per week, respectively. Additionally, the frequency of pain in the upper middle part of the stomach ranged from never (31.1%) to four to seven days per week (13.7%). The frequency of nausea ranged from never (39.6%) to four to seven days per week (6.8%). Finally, 59.0% of the participants reported taking medications for heartburn and regurgitation more than prescribed. The frequency of heartburn and regurgitation symptoms is shown in Table 5.

**Table 5:** GERD questionnaire demonstrating the frequency of heartburn and regurgitation symptoms among the study participants

N=293	0 days	1 day/week	2-3 days/week	4-7 days/week
How often do you have a burning feeling behind your breastbone (heartburn)?	94 (32.1%)	78 (26.6%)	76 (25.9%)	45 (15.4%)
How many times have the contents of your stomach (food or drink) moved upwards into your throat or mouth (regurgitation)?	70 (23.9%)	100 (34.1%)	80 (27.3%)	43 (14.7%)
How many times have you felt pain in the upper middle part of your stomach?	91 (31.1%)	77 (26.3%)	85 (29%)	40 (13.7%)
How often did you have nausea?	116 (39.6%)	88 (30%)	69 (23.5%)	20 (6.8%)
How often did you have difficulty getting a good night's sleep because of your heartburn and/or regurgitation?	89 (30.4%)	103 (35.2%)	76 (25.9%)	25 (8.5%)
How often did you take additional medication for your heartburn and/or regurgitation other than what the physician told you to take?	173 (59%)	53 (18.1%)	32 (10.9%)	35 (11.9%)

## Discussion

GERD and IBS are both debilitating diseases that affect a large number of individuals around the world [3,8]. Earlier, these were considered separate diseases, but similarities in their clinical manifestations and symptoms have led to the possibility of a disease co-occurrence. Our findings revealed that the co-occurrence between GERD and IBS was 35.8%. These results are concurrent with several studies worldwide and in Gulf countries showing a significant co-occurrence between GERD and IBS [9-12]. GERD and IBS affect gastrointestinal motility [9,11] and can be classified as functional dyspepsia, which causes gastrointestinal upset, but the causative factor is not fully understood [21,22]. However, a systematic review reported varied frequencies of co-occurrence between GERD and IBS, ranging between 1.7% and 74.7% [18]. Another study showed a 34% co-occurrence of GERD among IBS patients, whereas IBS co-occurred with GERD in 52% of cases [23]. Moreover, in another study conducted in the Netherlands on 263 GERD patients, 35% of them had IBS [24]. Interestingly, when IBS diagnosis was considered according to Rome IV criteria in our sample, the prevalence rates dropped to 3.8%. When the prior Rome II criteria were used, the co-occurrence between GERD was 14.2% and 26.7% when the Manning criteria were used [25]. Similarly, another study on IBS reported that when Rome III criteria were used, IBS prevalence was 33.8% [3].

In this context, GERD-IBS denotes the presence of IBS alongside pre-existing GERD, while IBS-GERD signifies the reverse scenario. In our study, GERD, IBS, and co-occurrence of GERD-IBS or IBS-GERD reduce the quality of life, in which the GERD-HQRL median was 16 and 15 for GERD with IBS co-occurrence and IBS with GERD co-occurrence, respectively. This study's results are similar to a study that found that 46.9% of patients with GERD-IBS and 34.4% of patients with IBS-GERD had lower health-related quality of life (HR-QOL) than the control population [26]. Similarly, a Taiwanese study found that GERD patients who have IBS had more severe GERD symptoms compared with patients with GERD or IBS alone [27].

Another study in the Danish population revealed that individuals exhibiting concurrent gastrointestinal symptom complexes show a markedly increased likelihood of reporting suboptimal self-assessed health and compromised functional ability compared to individuals presenting symptoms consistent with a single symptom complex [28]. Our study found that 41% of the participants had to take medication for gastric discomfort. This concomitates a study conducted on the Japanese population that revealed that both GERD and IBS, individually or in conjunction, were associated with abnormal stool patterns. In the co-occurrence group, 52.4% of the patients used medication for abnormal stool patterns and gastric discomfort [29].

Our study also found that 16.7% of the patients smoked; however, we did not find any association between smoking and the co-occurrence of GERD and IBS. In contrast to a previous study that found 39.3% of the patients had a co-occurrence of IBS and GERD, positively associated with smoking and increased age [7]. Additionally, the co-occurrence of diseases was found in 15% of the population, with 9.3% having uninvestigated dyspepsia and IBS, 8.6% showing symptoms of uninvestigated dyspepsia and GERD, and 4.6% having IBS and GERD [30]. This contrasts with our study, in which we found the co-occurrence of IBS and GERD was 38.5%. These differences may arise as different ethnicities have different incidences of these diseases [9].

The participants in the present study showed that 29.4% had recurrent abdominal pain, and 53.6% of patients reported a change in bowel movement experienced with the pain, decreasing their quality of life. This finding is in concurrence with a cross-sectional study done on the general population of Saudi Arabia about functional dyspepsia that found that the prevalence of IBS-like symptoms among dyspeptic patients was 44%, and these patients have the highest frequency of co-occurring symptoms (functional heartburn) and IBS, thus decreasing their quality of life due to persistent symptoms [31]. Our study reported that only 16.7% of participants were smokers. However, there are similarities in the percentage of respondents who exercised: 15.40% vs. 16.4% in the previous study [25]. In contrast, another study done on students in Saudi Arabia found that the prevalence of GERD among patients who are diagnosed with IBS is 66.7%; 72.8% were smokers, and only 10.1% of them exercised regularly [32]. It has become clear that the co-occurrence of GERD and IBS and vice versa is common, and our study explores the percentage of the population affected by these debilitating diseases and their impact on patients' lives. This study will help guide clinicians to classify the disease better, which will help implement preventive measures, personalized medical interventions, and thus better patient outcomes.

Limitations

One of the potential limitations of the study was that there was an over-labeling of participants as GERD patients in our sample compared to the GERD questionnaire diagnosis, but in the analysis, we excluded any GERD patient who did not meet the criteria in the GERD diagnosis questionnaire to prevent overestimation of the co-occurrence between IBS and GERD. In addition, our study utilized patient recollection as a means of gathering health history, a method that is prone to recall bias. The results' applicability to a larger population is still being determined due to the exclusive inclusion of participants from a singular institution.

## Conclusions

About 35.8% of GERD patients have IBS. Patients with both disorders had a considerably worse quality of life (high GERD-HRQL score). Healthcare practitioners should screen for both illnesses. Many participants reported taking more medications for heartburn and regurgitation than prescribed, underscoring the importance of assessing patients' quality of life when prescribing medications for GERD. Addressing lifestyle factors such as smoking and stress may also help manage symptoms. To understand the association between GERD and IBS, address its influence on quality of life, and create appropriate management options that account for regional cultural and environmental variables, more study is needed.
